# Attraction to Smelly Food in Birds: Insectivorous Birds Discriminate between the Pheromones of Their Prey and Those of Non-Prey Insects

**DOI:** 10.3390/biology10101010

**Published:** 2021-10-08

**Authors:** Luisa Amo, Irene Saavedra

**Affiliations:** 1 Departamento de Ecología Evolutiva, Museo Nacional de Ciencias Naturales (MNCN-CSIC), C/José Gutiérrez Abascal, 2, E-28006 Madrid, Spain; irene.saavedra.garces@gmail.com; 2 Area of Biodiversity and Conservation, Universidad Rey Juan Carlos, c/Tulipán s/n., E-28933 Madrid, Spain

**Keywords:** avian olfaction, foraging, insect pheromones, insectivorous birds, predator-prey interactions, prey chemical cues

## Abstract

**Simple Summary:**

The role of olfaction in avian life histories has traditionally been neglected, but a growing body of evidence suggests that birds use olfaction in different biological contexts, including foraging. Insectivorous birds are known to detect the defence volatiles emitted by trees when attacked by herbivore arthropods. Recently, it has been shown that insectivorous birds not only use these indirect cues to locate their prey but that they can also eavesdrop on the pheromones emitted by the prey. However, the questions of whether avian attraction is limited to prey pheromones only or whether they can detect any insect pheromone remain unexplored. Therefore, we performed a field experimental study using artificial larvae close to pheromone dispensers placed on trees to analyze whether birds are attracted to prey pheromones but not to non-prey pheromones or control unscented dispensers. We recorded the number of trees that contained artificial larvae with signals of avian predation and found that insectivorous birds were only attracted to prey pheromones, confirming that insectivorous birds are able to eavesdrop on prey pheromones and suggesting that birds are not attracted to non-prey pheromones.

**Abstract:**

Natural selection has favored the evolution of different capabilities that allow animals to obtain food—e.g., the development of senses for improving prey/food detection. Among these senses, chemical sense is possibly the most ancient mechanism used by organisms for environmental assessment. Comparative studies suggest the prime role of foraging ecology in the evolution of the olfactory apparatus of vertebrates, including birds. Here, we review empirical studies that have shown birds’ abilities to detect prey/food via olfaction and report the results of a study aiming to analyze the specificity of eavesdropping on prey pheromones in insectivorous birds. In a field study, we placed artificial larvae and a dispenser with one of three treatments—prey (*Operopthera brumata*) pheromones, non-prey (*Rhynchophorus ferrugineus*) pheromones, or a control unscented dispenser—on the branches of Pyrenean oak trees (*Quercus pyrenaica*). We then measured the predation rate of birds on artificial larvae. Our results show that more trees had larvae with signs of avian predation when they contained a prey pheromone dispenser than when they contained a non-prey pheromone dispenser or an unscented dispenser. Our results indicate that insectivorous birds can discriminate between the pheromones emitted by their prey and those emitted by non-prey insects and that they only exhibit attraction to prey pheromones. These results highlight the potential use of insectivorous birds in the biological control of insect pests.

## 1. Introduction

The olfactory system is one of the main sensory systems used by most animals [1], perhaps because chemical communication is the most ancient form of communication [1]. Despite the fact that the role of olfaction in avian life histories has traditionally been neglected, a growing body of evidence suggests that birds use olfaction in different biological contexts. At the intraspecific level (see [2,3] for reviews), evidence has shown that birds use olfaction to recognize their nest [4,5,6] and eggs [7]. Chemical cues also seem to play a role in parent [8,9] and sibling recognition [10]. Olfactory cues also seem to play a role in partner recognition [11], rival assessment [12], and mate choices [2,13]. Birds can discriminate the sex of conspecifics through olfaction [14,15,16]. Olfactory cues also allow birds to assess the similarity or dissimilarity of conspecifics in the Mayor Histocompability Complex (MHC) [7,17]. At the interspecific level, olfaction also seems to be useful for predation risk assessment [18,19,20,21,22,23,24,25], for the selection of aromatic plants used in nests [26,27,28,29], and for orientation and navigation [30,31,32].

Previous evidence also suggests that birds use olfaction in the process of foraging. The use of olfaction during foraging seems to be an ancient trait in birds (e.g., Kiwis (*Apteryx australis*) [33]; *Cathartes* vultures [34]), where it is maintained in modern lineages (Procellariiforms [35]); penguins [36,37]; domestic chickens (*Gallus gallus*) [38]; and passeriformes, such as zebra finches (*Taeniopygia guttata*) [39], great tits (*Parus major*), and blue tits (*Cyanistes caeruleus*) [40,41,42]). The results of a recent comparative study suggest that the olfactory bulb size, a proxy of olfactory capability, varies substantially across bird species in relation to diet type and ecological conditions [43], suggesting that foraging mode has played an important role in the evolution of chemosensory abilities in birds.

The use of chemical information in foraging activities could be an advantageous strategy by which to locate prey in places where visual information is only available at very short distances, such as in covered areas with dense vegetation. The role of bird olfaction in foraging contexts has been demonstrated in the kiwi (*Apteryx australis*) [33,44]. Additionally, vultures of the New World, such as turkey vultures (*Cathartes aura* [45]) and greater yellow-headed vultures (*C. melambrotus*) [46]), seem to use olfaction to locate the carcasses they feed on. The role of olfaction in foraging has also been suggested in honey-guides (family Indicatoridae [47]) and honey buzzards (*Pernis orientalis* [48]). The yellow-backed chattering lory (*Lorius garrulus* subsp. *flavopalliatus*) can distinguish one dispenser with artificial nectar from another with water through the detection of the essences of different plants [49]. In relation to fruit scent detection, the Kakapo (*Strigops habroptilus* also uses olfaction to find food [50], while house finches (*Carpodacus mexicanus*) exhibit a preference for an artificial red fruit scent [19].

Among the most interesting predator–prey relationships mediated by chemical cues are those involving several trophic levels. In the sea, Procellariiformes and Sphenisciformes can detect dimethyl sulphide (DMS [36,37]), a chemical compound released by phytoplankton when zooplankton and pelagic fishes graze on phytoplankton, thus signaling areas of high productivity in oceans [35,51]. Chemical communication also plays an important role in plant–herbivory–predator interactions in terrestrial systems, as insectivorous birds exploit the chemical indirect cues emitted by plants (Herbivore Induced Plant Volatiles, HIPVs) in response to caterpillar herbivory ([40,42,52,53,54,55,56,57], reviewed in [41]). When attacked by herbivorous predators, plants respond in a defensive manner to face such herbivory, including the emission of HIPVs that attract the predators of herbivores [58]. This herbivore-induced defence, called ‘crying for help’, has mainly been studied in relation to insects that prey upon larvae [59,60]. However, Mäntylä and collaborators provided the first evidence that insectivorous birds were attracted to herbivore-infested trees even when they could not see the larvae or their damage on the leaves [54]. Vision [52] or olfaction [53] have been proposed as the mechanisms responsible for this attraction, as larvae-infested trees differ from uninfected trees both in the reflectance of the leaves and in the HIPVs that they emit [40,52,53,55]. Amo and collaborators [40] isolated the visual and chemical cues of larvae-infested apple trees and found that great tits were attracted to infested trees when they could only smell but not when they could only see the trees. Therefore, it seems that HIPVs can attract insectivorous birds [40,42], although a previous study using artificial mixtures of volatiles has not found such an attraction [61]. The positive correlation found between avian the predation rates of artificial larvae and the quantity of emission of volatiles emitted by trees experimentally infested with caterpillars in natural conditions also suggests that olfaction may be the mechanism underlying bird attraction to caterpillar-infested trees [53], although vision may also play an important role in finding prey [42,52,57]. Avian attraction has also been shown in plants treated with methyl jasmonate (MeJA) [41,56], a phytohormone involved in the development of plant defence against herbivory [62,63,64]. However, in other plant–herbivore systems, no such avian attraction to MeJA-treated trees was found [65,66]. This variability in the response of birds may have been caused by the differences in the volatiles emitted by trees between herbivore-infected trees and those treated with MeJA [41]. Birds such as great tits and blue tits are also attracted to chemical cues released by Scots pines during pine sawfly oviposition [67]. This evidence shows that birds not only use herbivory-induced plant changes to detect larvae but that they also responsive to changes induced by insect oviposition to detect the insect clutch they feed on [67].

Birds not only use indirect cues to find their prey (see [41,51] for a review) but are also able to detect the chemical cues emitted by the prey itself. Insect species release pheromones to attract mates or other conspecifics [68,69]. However, while chemical signals such as pheromones can be detected by conspecifics, they can also be eavesdropped on by predators and parasites, increasing the risk of predation or parasitism for prey [70,71]. Many predator species can eavesdrop on the chemical cues involved in mate attraction or signaling in different taxa from invertebrates to vertebrates, such as insects, amphibians, reptiles, and mammals [1]. However, until recently, we were not aware that insectivorous birds could use olfaction to detect the pheromones of adult lepidopteran and use these to locate their prey. In a previous study, we found that insectivorous birds were able detect the pheromones emitted by winter moth (*Operophtera brumata*) females to attract males, exploiting these pheromones as a method of prey location to maximize their foraging effort [72]. However, the question of whether avian attraction to pheromones is exclusive to prey pheromones or it is exhibited toall insect pheromones remains to be answered. Here, we present the results of a study aimed to disentangle whether insectivorous birds can discriminate between the pheromones of one of their prey species from the pheromones of a non-prey insect.

## 2. Material and methods

### 2.1. Study Area and Species

This experimental study was carried out between July and August 2020 in a Pyrenean oak (*Quercus pyrenaica*) forest in Madrid province (Sierra de Guadarrama, Central Spain, 40°43′ N, 03°55′ W). In this forest, a population of insectivorous birds breeding in 100 wooden nest-boxes was established in 2017. The nest-boxes were occupied mainly by breeding pairs of blue tits (*C. caeruleus*) and some pairs of great tits (*P. major*). Other insectivorous bird species were observed in the study area at lower densities, including the common blackbird (*Turdus merula*), coal tit (*Periparus ater*), and Eurasian nuthatch (*Sitta europaea*). Tits feed mainly on caterpillars, such as *O*. *brumata*, during their breeding period [73]. However, during the winter, when no caterpillars are available, parids such as great tits and blue tits prey upon species belonging to the Hemiptera, Coleoptera, Hymenoptera, and Lepidoptera orders [73], such as *O*. *brumata* adults [73,74]. Thus, we chose this species as a prey species because *O*. *brumata* adults constitute an important part of the diet when they are available in winter. Furthermore, the results of a previous study have shown that insectivorous birds are attracted to the *O. brumata* pheromone [72]. *O*. *brumata* adults are present in the study area from November to February [75]. In this species, only females produce pheromones during the reproductive period to attract males [76,77]. We performed the study in July and August, outside of the reproductive period of this species (from November to February [75]), to ensure that adult moths were absent and therefore that bird attraction to the *O*. *brumata* pheromone could be attributed to the pheromone and not to the presence of males. The results of a previous study performed in a *Q. pyrenaica* forest after the period of emergence of *O. brumata* adults showed that traps baited with the pheromone did not attract *O. brumata* individuals or any insect species that could have been attracted by the same pheromone (e.g., predators or parasitoids) [72]. The pheromone of *O*. *brumata* is composed of 1,Z3,Z6,Z9-nonadecatetraene [76,77]. A synthetic version of this pheromone can be obtained from commercial suppliers (OPENNATUR, S.L.). Pheromone dispensers contain 0.5 mg of 1,Z3,Z6,Z9-nonadecatetraene (Pherobank, B.V.). The emission of this pheromone lasts 40 days, and thus the emission rate is approximately 9 ng/min (Pherobank, B.V.).

As a non-prey species, we chose to use the palm weevil, *Rhynchophorus ferrugineus*, which is a coleopteran species that is not present in the study area. The palm weevil is originally from tropical Asia but has spread to Europe, reaching the Mediterranean in the 1980s. However, because *R. ferrugineus* only attacks palms that belong to Arecales order, it is not present in the study area. The pheromone dispensers contain a blend of 4 Methyl 5 Nonanol (90%) and 4 Methyl 5 Nonanone (10%). The pheromone release rate was estimated to be 3 mg/day at 20 °C. The emission lasted 90 days and the emission rate was approximately 2 ng/min (Pherobank, B.V.).

### 2.2. Experimental Design and Procedure

**Figure 1:**
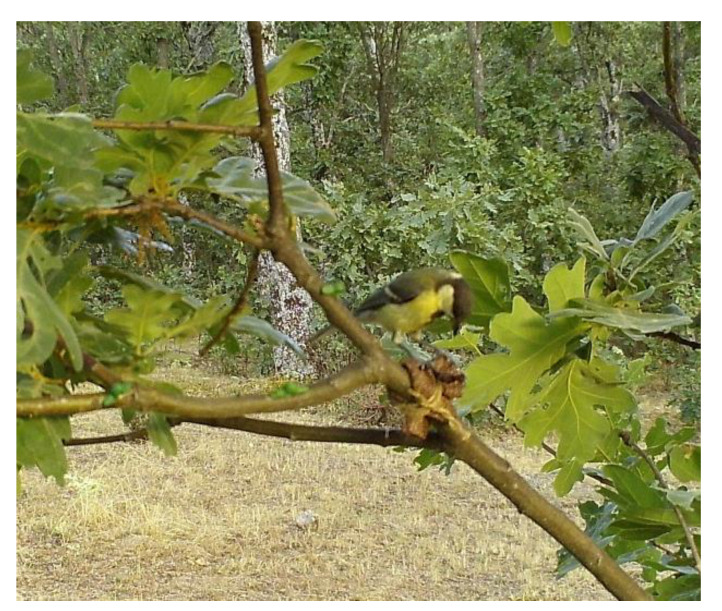
Photograph of a wild great tit, *Parus major*, on an *Operopthera brumata* pheromone dispenser hidden inside a raffia bag.

We placed experimental and control dispensers on branches of 75 Pyrenean oak trees. The branches were approximately 1.5 m long, with no evident signs of herbivory. The dispensers were placed at similar average heights in the trees (approx. 1.5 m high). Each dispenser was hidden inside a raffia bag that was fixed to the branch with a rope. Five artificial larvae were placed in the surroundings of the dispenser, at distances of 2 to 50 cm from the dispenser. Thus, the dispensers were situated in the middle of 5 artificial larvae. For the prey pheromone treatment, we placed an *O. brumata* pheromone dispenser inside the raffia bag. In the non-prey pheromone dispenser, we placed a *R. ferrugineus* pheromone dispenser inside the raffia bag. For the control treatment, we placed an unscented dispenser similar to that of the *O. brumata* pheromone dispenser that was also hidden inside a raffia bag. Therefore, all the treatments were visually similar (Figure 1). The artificial larvae were made of light green plasticine (similar to the natural color of real *O. brumata* larvae, at least to a human’s visual perception). Neither the plasticine caterpillars nor the raffia bags emitted UV light. The plasticine larvae were approximately the size of a large fifth instar *O. brumata* larva (length 25 ± 30 mm, Ø 3 ± 4 mm). The plasticine larvae were attached with cyanoacrylate adhesive glue to the branches of forest oak trees. Experimental trees were separated by gaps of at least 40 m. The trees were alternatively assigned to one of the treatments: commercial *O. brumata* pheromone dispenser (prey pheromone, n = 25), commercial *R. ferrugineus* pheromone dispenser (non-prey pheromone, n = 25), or odorless control dispenser (control, n = 25). Thus, the treatments were spatially intermixed among the oak trees.

To study the attraction of the insectivorous birds to the pheromones, we checked the number of larvae in the trees with predation marks made by birds every 2 days over a period of 13 days. Artificial caterpillar models have previously been used to estimate insectivorous bird attraction [53,62,72,78,79,80,81,82]. A predation event was assigned to a tree when the tree contained at least one larva damaged by birds. Larva models were considered damaged when they had triangle-shaped marks and deep cuts made by the beaks of birds and when a part of their body was taken by the birds, as described in Mäntylä and collaborators [53,66]. Each model showing a predation mark was replaced with a new one at the same location during the visits, as we counted the number of larvae with signs of avian predation each visit.

The treatments were in place for 13 days, a period of time for which the effectiveness of the commercial pheromones is guaranteed, as they can last up to 40 days for the *O. brumata* pheromone and 90 days for the *R. ferrugineus* pheromone (Pherobank, B.V.). We placed a BROWNING DARK OPS HD camera trap close to a tree containing a prey pheromone dispenser for two days to gain an idea of how birds approach the experimental area and attempt to prey on the artificial caterpillars. At the end of the experiment, we removed all plasticine larvae and the commercial pheromones and controls. The experiment was conducted under a license issued by the Dirección General de Biodiversidad y Recursos Naturales, Consejería de Medio Ambiente, Ordenación del Territorio y Sostenibilidad, Comunidad de Madrid (Ref. 10/024906.9/20).

### 2.3. Statistical Analyses

We modelled the probability that at least one predation event would occur in a tree in relation to the treatment (prey pheromone vs. non-prey pheromone vs. control) with a generalized linear model (GLM) fit using the Laplace approximation with binomial errors and a logit link function. We also analyzed the probability that at least one event would occur per visit in relation to treatment with a generalized linear model (GLM) fit using the Laplace approximation with binomial errors and a logit link function. Data analyses were performed with the statistical program R 4.0 stats package [83].

## 3. Results

**Figure 2:**
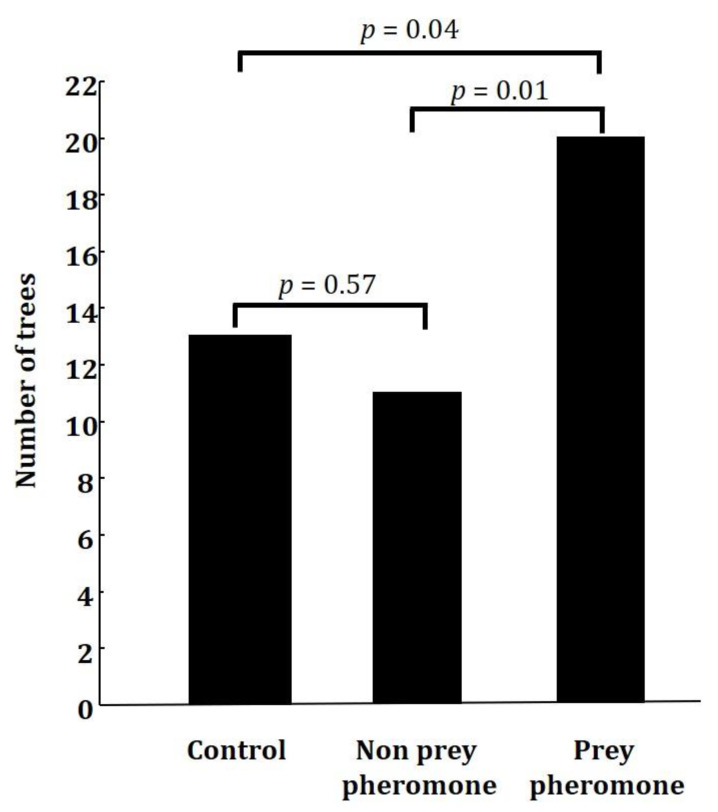
Number of trees with at least one artificial larva with marks of avian predation when the tree contained a prey (*Operophtera brumata*) pheromone dispenser (n = 45), a non-prey (*Rhynchophorus ferrugineus*) pheromone dispenser (n = 45), or a control unscented dispenser (n = 45).

The number of trees that had at least one caterpillar with signs of avian predation differed between treatments (GLM: χ^2^ = 7.77, df = 2, *p* = 0.02, Figure 2). Post hoc comparisons showed that the differences were significant when comparing the prey pheromone treatment and the control treatment (*p* = 0.04) and the prey and non-prey treatments (*p* = 0.01). There were no differences in the number of trees that had at least one caterpillar with signs of avian predation between the non-prey and the control treatments (*p* = 0.57). Twenty out of the 25 trees containing a prey pheromone dispenser had at least one avian predation event (i.e., at least one artificial caterpillar had signs of avian predation). In contrast, a predation event was observed in only 13 out of the 25 control trees and in 11 out of 25 trees containing the non-prey pheromone dispenser. The proportion of visits where a predation event was detected also differed between treatments (GLM: χ^2^ = 17.76, df = 2, *p* = 0.0001). Post hoc comparisons showed that the differences were significant when comparing the prey pheromone treatment and the control treatment (*p* = 0.01) and the prey treatment and non-prey treatment (*p* < 0.0001). There were no differences in the number of trees that had at least one caterpillar with signs of avian predation between the non-prey pheromone treatment and the control treatment (*p* = 0.08).

## 4. Discussion

Our results confirm that insectivorous birds can eavesdrop on the pheromones emitted by their prey and suggest that this attraction is only towards prey pheromones and not towards non-prey pheromones. The number of trees that contained at least one artificial caterpillar with signals of predation by birds was higher when the trees contained a prey (*O. brumata*) pheromone dispenser than when they contained a non-prey (*R. ferrugineus*) or a control unscented dispenser. Furthermore, we found at least one artificial larva predated in a greater number of visits when trees had a prey pheromone dispenser than when they had a non-prey pheromone dispenser or a control dispenser.

We performed this study during the summer when there are no adults of *O. brumata*. The adults of this species emerge in November and can be observed in the field until February [75]. Therefore, the greater predation rates of artificial larvae cannot be due to the attraction of birds to the presence of *O. brumata* males close to the female pheromone, as previously demonstrated with moth traps baited with this pheromone [72].

Therefore, our results suggest that insectivorous birds can eavesdrop on the intraspecific cues emitted for mate attraction, as has been observed in other taxa, and that this attraction is exclusively exhibited towards prey chemical cues. The photographs obtained by the trap camera also confirm that birds are guided by the scent released by the pheromone dispenser, as we observed a great tit on the cotton bag containing the dispenser, first pecking at the bag (Figure 1) and then paying attention to the artificial caterpillars. We also found several bags containing a prey pheromone dispenser that were attacked by birds. This behavior suggests that birds use pheromones as a precise localization cue. The emission rate of prey pheromone dispensers has been proven to be biologically relevant, as both *O. brumata* males and insectivorous birds [72] are attracted to them. However, this emission rate is similar to the emission rate of 10 *O. brumata* females [84], suggesting that birds are attracted to trees where they can find several females. Therefore, further studies are needed to disentangle whether birds can detect the pheromone emission of a single female or whether they are just attracted to cues signaling areas with a high prey availability. We used commercial dispensers designed to attract *O. brumata* and *R. ferrugineus* to traps. Both dispensers differed in their emission rate, with the emission rate of the *O. brumata* pheromone dispenser being approximately 9 ng/min, whereas the emission rate of *R. ferrugineus* was approximately 2 ng/min. Although the dispensers were designed to attract conspecifics of *O. brumata* and *R. ferrugineus*, and predators may detect similar concentrations of pheromones as their prey, the lower emission rate of the non-prey pheromones dispenser may have caused birds to not be able to detect this scent from as far as the prey pheromone dispenser; therefore, this may explain the lack of attraction to non-prey pheromone dispensers. However, if the bird attraction was due just to differences in the emission rate of the dispensers, we would likely have found more caterpillars with signals of avian attacks in the non-prey pheromone treatment than in the control unscented treatment, which had an emission rate of 0. However, that lack of differences between the control treatment and the non-prey pheromone treatment (*p* = 0.57) suggests that insectivorous birds are attracted to the pheromone of a prey species but not to that of a non-prey species. In any case, further experiments using dispensers with similar emission rates are needed to confirm that the lack of attraction of avian predators towards non-prey pheromones is not due to the lower emission rate of these dispensers.

We chose to use *R. ferrugineus* as a non-prey insect because we aimed to ensure that the pheromone did not attract individuals to the dispensers. However, as *R. ferrugineus* is a foreign species, our results do not allow us to disentangle whether insectivorous birds are not attracted to *R. ferrugineus* because it is a non-prey species or because it is a foreign species. Therefore, further studies are needed to explore how experience helps insectivorous birds find their insect prey. Whether the ability to detect prey-related odors is innate or acquired through experience may depend on the degree of specialization of predators as well as on the variability of prey-associated chemical cues. These two factors may explain why, even when birds use indirect chemical cues that signal the presence of prey, both innate and learned responses can be found in the literature [85,86]. Innate recognition of food-related odors (DMS) has been found in Procelariiform birds [85]. DMS is a compound released by phytoplankton when attacked that signals areas of high productivity in the oceans where Procellarifom seabirds can find their prey. In contrast, previous evidence suggests that insectivorous birds that use HIPVs to locate their prey need to learn to associate HIPVs with a foraging experience [86,87]. HIPVs are highly variable, as their composition depends not only on the herbivore species involved but also on the plant species [41]. Insectivorous birds can feed on different prey species, which feed on different tree species, which release different blends of HIPVs. Therefore, in these generalist predators exposed to such a high variability of HIPVs, the ability to learn to associate a foraging experience with the presence of such indirect chemical cues has been favored over an innate recognition of different volatiles [86,87], as has been observed in predator–prey interactions in insects [88,89]. However, to the best of our knowledge, no studies have examined whether the detection of such direct chemical cues is innate or may be learned. A potential explanation for the lack of attraction to non-prey pheromones could be that insectivorous birds that are naïve to this scent exhibited an aversive response to it. Although birds can exhibit neophobic responses to some scents [90], in other studies with insectivorous birds we have not found an aversive response to unknown scents, even inside the nest cavity [18]. Furthermore, the results of our study showed that birds approached the non-prey dispenser similarly than the control one (*p* = 0.57), which indicates that possible processes of either, neophobia or neophylia towards the used non-prey scent unlikely affected our results. 

The attraction to chemical cues emitted by prey species has been previously demonstrated in several taxa, from invertebrates to vertebrates, such as insects, amphibians, reptiles, and mammals [1]. For example, olfactory cues emitted by small mammals are known to attract carnivores such as least weasels [91,92,93], cats, foxes, and snakes [94]. Chemical cues emitted by lizards are also detected by saurophagous snakes [95]. Therefore, the release of chemical cues for attracting partners increases the risk of detection by predators [71,96]. Furthermore, cats exhibit a preference for rats emitting high pheromone levels over those with low pheromone levels [97]. This result reveals the conflict between sexual attractiveness and predation risk, which may have modulated the evolution of these chemical signals [1].

In birds, attraction to cues emitted by prey has been previously shown in predatory raptor species, such as rough-legged buzzards (*Buteo lagopus*), common kestrels (*Falco tinnunculus*), and grey shrikes (*Lanius excubitor*), which are attracted to the urine marks of their small mammal prey. The visual detection of the UV coloration of urine has been proven to be the mechanism responsible for such detection [98,99,100]. However, whether these raptor species also use olfaction in foraging remains unexplored, but could be possible, as the use of olfaction in foraging has been demonstrated in different bird species. For example, kiwis use olfaction to locate small invertebrates on the ground, although the specific compounds involved in such detection remain unknown [33,44]. Great tits, blue tits, and willow warblers (*Phylloscopus trochilus*), are attracted to the defence volatiles emitted by trees infested by caterpillars [40,42,52,53,55] or by insect eggs [67].

The exclusive attraction to prey pheromones but not to non-prey pheromones points to the potential use of insectivorous birds for controlling defoliator caterpillar numbers in orchards and forests. Attraction to female pheromones may increase not only a bird’s probability of finding females but also the male moths that are attracted to this pheromone. Therefore, birds may be more efficient than pheromone traps in decreasing the number of Lepidopteran caterpillars [101]; thus, insectivorous birds can be considered excellent candidates for use as predators in the biological control of insect plagues [102,103].

## 5. Conclusions

Our results confirm that insectivorous birds use the pheromones emitted by their prey to locate them. Bird attraction to pheromones was not exhibited towards a non-prey pheromone, suggesting that birds are not attracted to all insect pheromones but exclusively eavesdrop on insect prey chemical cues. Aversive responses to new scents can be excluded, and the similar responses to unscented dispensers and non-prey pheromone dispensers suggests that the emission rate did not influence this result. Further experimental studies using similar emission rates for prey and non-prey pheromones are needed to rule out the possibility that the lack of attraction to non-prey pheromones could be caused by the lower emission rate of such dispensers.

## Data Availability

All data used in this manuscript are reported in the manuscript.

## References

[1] Wyatt T.D. (2003). Pheromones and Animal Behaviour: Communication by Smell and Taste.

[2] Caro S.P., Balthazart J., Bonadonna F. (2015). The perfume of reproduction in birds: Chemosignaling in avian social life. Horm. Behav..

[3] Whittaker D.J., Hagelin J.C. (2021). Female-Based Patterns and Social Function in Avian Chemical Communication. J. Chem. Ecol..

[4] Bonadonna F., Villafane M., Bajzak C., Jouventin P. (2004). Recognition of burrow’s olfactory signature in blue petrels, Halobaena caerulea: An efficient discrimination mechanism in the dark. Anim. Behav..

[5] Caspers B.A., Krause T. (2010). Odour-based natal nest recognition in the zebra finch (Taeniopygia guttata), a colony-breeding songbird. Biol. Lett..

[6] Krause E.T., Caspers B. (2012). Are Olfactory Cues Involved in Nest Recognition in Two Social Species of Estrildid Finches?. PLoS ONE.

[7] LeClaire S., Strandh M., Mardon J., Westerdahl H., Bonadonna F. (2017). Odour-based discrimination of similarity at the major histocompatibility complex in birds. Proc. R. Soc. B Boil. Sci..

[8] Caspers B.A., Hagelin J.C., Paul M., Bock S., Willeke S., Krause E.T. (2017). Zebra Finch chicks recognise parental scent, and retain chemosensory knowledge of their genetic mother, even after egg cross-fostering. Sci. Rep..

[9] Griebel I.A., Dawson R.D. (2020). Nestling tree swallows ( Tachycineta bicolor ) alter begging behaviour in response to odour of familiar adults, but not their nests. Ethology.

[10] Rossi M., Marfull R., Golüke S., Komdeur J., Korsten P., Caspers B.A. (2017). Begging blue tit nestlings discriminate between the odour of familiar and unfamiliar conspecifics. Funct. Ecol..

[11] Bonadonna F. (2004). Partner-Specific Odor Recognition in an Antarctic Seabird. Science.

[12] Amo L., López-Rull I., Pagán I., Garcia C.M. (2012). Male quality and conspecific scent preferences in the house finch, Carpodacus mexicanus. Anim. Behav..

[13] Hirao A., Aoyama M., Sugita S. (2009). The role of uropygial gland on sexual behavior in domestic chicken Gallus gallus domesticus. Behav. Process..

[14] Zhang J.-X., Wei W., Zhang J.-H., Yang W.-H. (2010). Uropygial Gland-Secreted Alkanols Contribute to Olfactory Sex Signals in Budgerigars. Chem. Sens..

[15] Whittaker D., Richmond K.M., Miller A.K., Kiley R., Burns C.B., Atwell J.W., Ketterson E.D. (2011). Intraspecific preen oil odor preferences in dark-eyed juncos (Junco hyemalis). Behav. Ecol..

[16] Amo L., Avilés J., Parejo D., Peña A., Rodríguez-Ruiz J., Tomas G. (2012). Sex recognition by odour and variation in the uropygial gland secretion in starlings. J. Anim. Ecol..

[17] Grieves L., Gloor G., Bernards M., MacDougall-Shackleton E. (2019). Songbirds show odour-based discrimination of similarity and diversity at the major histocompatibility complex. Anim. Behav..

[18] Amo L., Galván I., Tomás G., Sanz J.J. (2008). Predator odour recognition and avoidance in a songbird. Funct. Ecol..

[19] Amo L., López-Rull I., Pagán I., Garcia C.M. (2015). Evidence that the house finch (Carpodacus mexicanus) uses scent to avoid omnivore mammals. Rev. Chil. Hist. Nat..

[20] Amo L., Tomas G., López-García A. (2017). Role of chemical and visual cues of mammalian predators in nest defense in birds. Behav. Ecol. Sociobiol..

[21] Roth T.C., Cox J.G., Lima S.L. (2008). Can foraging birds assess predation risk by scent?. Anim. Behav..

[22] Eichholz M.W., Dassow J.A., Stafford J.D., Weatherhead P.J. (2012). Experimental evidence that nesting ducks use mammalian urine to assess predator abundance. Auk.

[23] Parejo D., Amo L., Rodriguez J., Aviles J.M. (2012). Rollers smell the fear of nestlings. Biol. Lett..

[24] Zidar J., Løvlie H. (2012). Scent of the enemy: Behavioural responses to predator faecal odour in the fowl. Anim. Behav..

[25] Saavedra I., Amo L. (2019). Egg concealment is an antipredatory strategy in a cavity-nesting bird. Ethology.

[26] Clark L., Mason J.R. (1987). Olfactory discrimination of plant volatiles by the European starling. Anim. Behav..

[27] Petit C., Hossaert-McKey M., Perret P., Blondel J., Lambrechts M.M. (2002). Blue tits use selected plants and olfaction to maintain an aromatic environment for nestlings. Ecol. Lett..

[28] Gwinner H., Berger S. (2008). Starling males select green nest material by olfaction using experience-independent and experience-dependent cues. Anim. Behav..

[29] Mennerat A. (2008). Blue tits (Cyanistes caeruleus) respond to an experimental change in the aromatic plant odour composition of their nest. Behav. Process..

[30] Wallraff H.G. (2004). Avian olfactory navigation: Its empirical foundation and conceptual state. Anim. Behav..

[31] A Nevitt G., Bonadonna F. (2005). Sensitivity to dimethyl sulphide suggests a mechanism for olfactory navigation by seabirds. Biol. Lett..

[32] Gagliardo A. (2013). Forty years of olfactory navigation in birds. J. Exp. Biol..

[33] Cunningham S.J., Castro I., Potter M. (2009). The relative importance of olfaction and remote touch in prey detection by North Island brown kiwis. Anim. Behav..

[34] Gomez L.G., Houston D.C., Cotton P., Tye A. (1994). The role of Greater Yellow-headed Vultures Cathartes melambrotus as scavengers in neotropical forest. Ibis.

[35] Nevitt G.A., Veit R.R., Kareiva P. (1995). Dimethyl sulphide as a foraging cue for Antarctic Procellariiform seabirds. Nat. Cell Biol..

[36] Cunningham G.B., Strauss V., Ryan P. (2008). African penguins (Spheniscus demersus) can detect dimethyl sulphide, a prey-related odour. J. Exp. Biol..

[37] Amo L., Rodríguez-Gironés M., Barbosa A. (2013). Olfactory detection of dimethyl sulphide in a krill-eating Antarctic penguin. Mar. Ecol. Prog. Ser..

[38] Marples N.M., Roper T.J. (1996). Effects of novel colour and smell on the response of naive chicks towards food and water. Anim. Behav..

[39] Kelly D.J., Marples N. (2004). The effects of novel odour and colour cues on food acceptance by the zebra finch, Taeniopygia guttata. Anim. Behav..

[40] Amo L., Jansen J.J., van Dam N.M., Dicke M., Visser M.E. (2013). Birds exploit herbivore-induced plant volatiles to locate herbivorous prey. Ecol. Lett..

[41] Mrazova A., Sam K., Amo L. (2019). What do we know about birds’ use of plant volatile cues in tritrophic interactions?. Curr. Opin. Insect Sci..

[42] Mäntylä E., Kipper S., Hilker M. (2020). Insectivorous birds can see and smell systemically herbivore-induced pines. Ecol. Evol..

[43] Avilés J.M., Amo L. (2017). The Evolution of Olfactory Capabilities in Wild Birds: A Comparative Study. Evol. Biol..

[44] Wenzel B.M. (1968). Olfactory Prowess of the Kiwi. Nat. Cell Biol..

[45] Houston D.C. (1986). Scavenging Efficiency of Turkey Vultures in Tropical Forest. Condor.

[46] Graves G.R. (1992). Greater Yellow-Headad Vulture (Cathartes melambrotus) Locates Food by Olfaction. J. Raptor Res..

[47] Stager K.E. (1967). Avian Olfaction. Am. Zool..

[48] Yang S.-Y., Walther B.A., Weng G.-J. (2015). Stop and Smell the Pollen: The Role of Olfaction and Vision of the Oriental Honey Buzzard in Identifying Food. PLoS ONE.

[49] Roper T.J. (2003). Olfactory discrimination in Yellow-backed Chattering Lories Lorius garrulus flavopalliatus: First demonstration of olfaction in Psittaciformes. Ibis.

[50] Hagelin J.C. (2003). Observations on the olfactory ability of the Kakapo Strigops habroptilus, the critically endangered parrot of New Zealand. Ibis.

[51] Nevitt G.A. (2011). The Neuroecology of Dimethyl Sulfide: A Global-Climate Regulator Turned Marine Infochemical. Integr. Comp. Biol..

[52] Mäntylä E., Klemola T., Sirkia P., Laaksonen T. (2007). Low light reflectance may explain the attraction of birds to defoliated trees. Behav. Ecol..

[53] Mantyla E., Alessio G.A., Blande J.D., Heijari J., Holopainen J.K., Laaksonen T., Piirtola P., Klemola T. (2008). From Plants to Birds: Higher Avian Predation Rates in Trees Responding to Insect Herbivory. PLoS ONE.

[54] Mantyla E., Klemola T., Haukioja E. (2004). Attraction of willow warblers to sawfly-damaged mountain birches: Novel function of inducible plant defences?. Ecol. Lett..

[55] Mäntylä E., Kleier S., Kipper S., Hilker M. (2016). The attraction of insectivorous tit species to herbivore-damaged Scots pines. J. Ornithol..

[56] Mrazova A., Sam K. (2017). Application of methyl jasmonate to grey willow (Salix cinerea) attracts insectivorous birds in nature. Arthropod-Plant Interact..

[57] Rubene D., Leidefors M., Ninkovic V., Eggers S., Low M. (2018). Disentangling olfactory and visual information used by field foraging birds. Ecol. Evol..

[58] Heil M. (2008). Indirect defence via tritrophic interactions. New Phytol..

[59] Takabayashi J., Dicke M., Posthumus M.A. (1991). Variation in composition of predator-attracting allelochemicals emitted by herbivore-infested plants: Relative influence of plant and herbivore. Chemoecology.

[60] Kessler A. (2001). Defensive Function of Herbivore-Induced Plant Volatile Emissions in Nature. Science.

[61] Koski T.-M., Laaksonen T., Mantyla E., Ruuskanen S., Li T., Girón-Calva P.S., Huttunen L., Blande J.D., Holopainen J.K., Klemola T. (2015). Do Insectivorous Birds use Volatile Organic Compounds from Plants as Olfactory Foraging Cues? Three Experimental Tests. Ethology.

[62] Hopke J., Donath J., Blechert S., Boland W. (1994). Herbivore-induced volatiles: The emission of acyclic homoterpenes from leaves of Phaseolus lunatus and Zea mays can be triggered by a β-glucosidase and jasmonic acid. FEBS Lett..

[63] Thaler J.S., Farag M.A., Paré P.W., Dicke M. (2002). Jasmonate-deficient plants have reduced direct and indirect defences against herbivores. Ecol. Lett..

[64] Thaler J.S., Stout M.J., Karban R., Duffey S.S. (1996). Exogenous jasmonates simulate insect wounding in tomato plants (Lycopersicon esculentum) in the laboratory and field. J. Chem. Ecol..

[65] Saavedra I., Amo L. (2018). Are wild insectivorous birds attracted to methyl-jasmonate-treated Pyrenean oak trees?. Behaviour.

[66] Mäntylä E., Blande J., Klemola T. (2014). Does application of methyl jasmonate to birch mimic herbivory and attract insectivorous birds in nature?. Arthropod-Plant Interact..

[67] Mäntylä E., Kleier S., Lindstedt C., Kipper S., Hilker M. (2018). Insectivorous Birds Are Attracted by Plant Traits Induced by Insect Egg Deposition. J. Chem. Ecol..

[68] Touhara K. (2008). Sexual communication via peptide and protein pheromones. Curr. Opin. Pharmacol..

[69] Yew J.Y., Chung H. (2015). Insect pheromones: An overview of function, form, and discovery. Prog. Lipid Res..

[70] Zuk M., Kolluru G.R. (1998). Exploitation of Sexual Signals by Predators and Parasitoids. Q. Rev. Biol..

[71] Hughes N.K., Kelley J.L., Banks P. (2012). Dangerous liaisons: The predation risks of receiving social signals. Ecol. Lett..

[72] Saavedra I., Amo L. (2018). Insectivorous birds eavesdrop on the pheromones of their prey. PLoS ONE.

[73] Betts M.M. (1955). The Food of Titmice in Oak Woodland. J. Anim. Ecol..

[74] Vel’Ký M., Kaňuch P., Krištín A. (2011). Food composition of wintering great tits (Parus major): Habitat and seasonal aspects. Folia Zool..

[75] Soria S. (1987). Operopthera brumata. Lepidopteros Defoliadores de Quercus Pyrenaica, Willdenow, 1805. Boletin de Sanidad Vegetal. Secretaría Técnica.

[76] Bestmann H.J., Brosche T., Koschatzky K.H., Michaelis K., Platz H., Roth K., Süβ J., Vostrowsky O., Knauf W. (1982). Pheromone-XLII. 1,3,6,9-nonadecatetraen, das sexualpheromon des frostspanners operophtera brumata (geometridae). Tetrahedron Lett..

[77] Roelofs W.L., Hill A.S., Linn C.E., Meinwald J., Jain S.C., Herbert H.J., Smith R.F. (1982). Sex Pheromone of the Winter Moth, a Geometrid with Unusually Low Temperature Precopulatory Responses. Science.

[78] Posa M.R.C., Sodhi N.S., Koh L.P. (2007). Predation on artificial nests and caterpillar models across a disturbance gradient in Subic Bay, Philippines. J. Trop. Ecol..

[79] Richards L.A., Coley P.D. (2006). Seasonal and habitat differences affect the impact of food and predation on herbivores: A comparison between gaps and understory of a tropical forest. Oikos.

[80] Remmel T., Davison J., Tammaru T. (2011). Quantifying predation on folivorous insect larvae: The perspective of life-history evolution. Biol. J. Linn. Soc..

[81] Tvardikova K., Novotny V. (2012). Predation on exposed and leaf-rolling artificial caterpillars in tropical forests of Papua New Guinea. J. Trop. Ecol..

[82] Sam K., Koane B., Novotny V. (2014). Herbivore damage increases avian and ant predation of caterpillars on trees along a complete elevational forest gradient in Papua New Guinea. Ecography.

[83] (2015). R: A Language and Environment for Statistical Computing. R Foundation for Statistical Computing.

[84] Jain S.C., Roelofs W.L., Meinwald J. (1983). Synthetic of sex attractant pheromone from a geometrid moth Operopthera brumata (the winter moth). J. Org. Chem..

[85] Bonadonna F., Caro S., Jouventin P., Nevitt G.A. (2006). Evidence that blue petrel, Halobaena caerulea, fledglings can detect and orient to dimethyl sulfide. J. Exp. Biol..

[86] Amo L., Dicke M., Visser M.E. (2016). Are naïve birds attracted to herbivore-induced plant defences?. Behaviour.

[87] Sam K., Kovarova E., Freiberga I., Uthe H., Weinhold A., Jorge L.R., Sreekar R. (2021). Great tits (Parus major) flexibly learn that herbivore-induced plant volatiles indicate prey location: An experimental evidence with two tree species. Ecol. Evol..

[88] Vet L.E.M., Dicke M. (1992). Ecology of Infochemical Use by Natural Enemies in a Tritrophic Context. Annu. Rev. Entomol..

[89] Gols R., Veenemans C., Potting R.P., Smid H.M., Dicke M., Harvey J.A., Bukovinszky T. (2012). Variation in the specificity of plant volatiles and their use by a specialist and a generalist parasitoid. Anim. Behav..

[90] Roper T.J. (1999). Olfaction in Birds. Adv. Study Behav..

[91] Cushing B.S. (1984). A selective preference by least weasels for oestrous versus dioestrous urine of prairie deer mice. Anim. Behav..

[92] Cushing B.S. (1985). Estrous Mice and Vulnerability to Weasel Predation. Ecology.

[93] Ylönen H., Sundell J., Tiilikainen R., Eccard J.A., Horne T. (2003). Weasels’ (Mustela Nivalis Nivalis) Preference for Olfactory Cues of The Vole (Clethrionomys Glareolus). Ecology.

[94] Hughes N.K., Price C., Banks P. (2010). Predators Are Attracted to the Olfactory Signals of Prey. PLoS ONE.

[95] Amo L., López P., Martin J. (2004). Chemosensory Recognition of Its Lizard Prey by the Ambush Smooth Snake, Coronella austriaca. South Am. J. Herpetol..

[96] Basolo A.L., Wagner W.E. (2004). Covariation between predation risk, body size and fin elaboration in the green swordtail, Xiphophorus helleri. Biol. J. Linn. Soc..

[97] Zhang Y.-H., Liang H.-C., Guo H.-L., Zhang J.-X. (2016). Exaggerated male pheromones in rats may increase predation cost. Curr. Zool..

[98] Viitala J., Korplmäki E., Palokangas P., Koivula M. (1995). Attraction of kestrels to vole scent marks visible in ultraviolet light. Nat. Cell Biol..

[99] Probst R., Pavlicev M., Viitala J. (2002). UV reflecting vole scent marks attract a passerine, the great grey shrike Lanius excubitor. J. Avian Biol..

[100] Zampiga E., Gaibani G., Csermely D., Frey H., Hoi H. (2006). Innate and learned aspects of vole urine UV-reflectance use in the hunting behaviour of the common kestrel Falco tinnunculus. J. Avian Biol..

[101] McNeil J.N. (1991). Behavioral Ecology of Pheromone-Mediated Communication in Moths and its Importance in the use of Pheromone Traps. Annu. Rev. Entomol..

[102] Mäntylä E., Klemola T., Laaksonen T. (2010). Birds help plants: A meta-analysis of top-down trophic cascades caused by avian predators. Oecologia.

[103] Mols C.M.M., Visser M.E. (2002). Great tits can reduce caterpillar damage in apple orchards. J. Appl. Ecol..

